# Association of the Endothelial Nitric Oxide Synthase (eNOS) G894T Gene Polymorphism With Type 2 Diabetes Mellitus in a Tunisian Population

**DOI:** 10.1002/edm2.70122

**Published:** 2025-12-10

**Authors:** Afif Ba, Manel Ayoub, Sana Aboulkacem, Mariem Belhedi, Zied Aouni, C. Mazigh

**Affiliations:** ^1^ Biochemistry Department Main Military Hospital of Tunis Tunis Tunisia; ^2^ Faculty of Pharmacy Monastir Tunisia; ^3^ Faculty of Medicine Tunis Tunisia; ^4^ Laboratory Department Habib Thameur Hospital Tunis Tunisia

**Keywords:** endothelial dysfunction, eNOS, G894T polymorphism, genetic association, nitric oxide, *NOS3*, type 2 diabetes

## Abstract

**Background:**

Type 2 Diabetes Mellitus (T2D) is a multifactorial metabolic disorder with a significant genetic component. Endothelial dysfunction, characterised by reduced nitric oxide (NO) bioavailability, is a key pathological feature. The endothelial nitric oxide synthase (eNOS) gene (*NOS3*) contains several polymorphisms, with the G894T (Glu298Asp) variant being a prominent candidate for influencing disease susceptibility.

**Objective:**

This study aimed to investigate the association between the eNOS G894T polymorphism and the risk of T2D in a sample of the Tunisian population.

**Methods:**

We conducted a case–control study including 100 T2D patients and 100 non‐diabetic controls recruited from the Military Hospital of Tunis. Anthropometric, clinical and biochemical parameters, including lipid profiles and high‐sensitivity C‐reactive protein (CRPus), were measured. Genotyping of the eNOS G894T polymorphism was performed using the Polymerase Chain Reaction–Restriction Fragment Length Polymorphism (PCR‐RFLP) method with the *Ban*II restriction enzyme.

**Results:**

T2D patients exhibited significantly higher levels of triglycerides (1.99 ± 1.27 vs. 1.45 ± 0.65 mmol/L, *p* = 0.002) and CRPus (2.73 ± 2.47 vs. 1.63 ± 1.42 mg/L, *p* = 0.003) compared to controls. The frequency of the mutated T allele was significantly higher in the T2D group than in the control group (27.53% vs. 11.27%, *p* < 10^−3^). Consequently, the heterozygous GT genotype was more prevalent among patients (55.07% vs. 19.72%, *p* < 10^−3^). The presence of the T allele was associated with a significantly increased risk of T2D (Odds Ratio [OR] = 4.495, 95% Confidence Interval [CI] = 2.14–9.44).

**Conclusion:**

The eNOS G894T polymorphism is a significant genetic risk factor for type 2 diabetes in the studied Tunisian population. The T allele appears to confer susceptibility, likely through mechanisms involving impaired eNOS function, reduced NO production and subsequent endothelial dysfunction.

## Graphical abstract

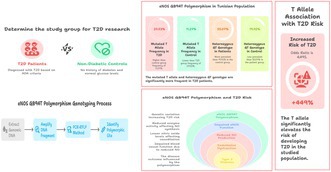

## 1 Introduction

Type 2 Diabetes Mellitus (T2D) has emerged as a global public health crisis, with its prevalence rising in parallel with obesity and sedentary lifestyles [1, 2]. T2D is a complex metabolic disease characterised by chronic hyperglycemia resulting from insulin resistance and relative insulin deficiency. Its severity is compounded by long‐term microvascular (retinopathy, nephropathy) and macrovascular (cardiovascular disease) complications [3].

The aetiology of T2D is multifactorial, arising from a complex interplay between environmental factors and genetic predisposition. A critical pathophysiological component of T2D and its complications is endothelial dysfunction, which is often an early event in the development of insulin resistance [4]. The endothelium plays a vital role in vascular homeostasis, primarily through the synthesis of nitric oxide (NO), a potent vasodilator with anti‐thrombotic, anti‐proliferative and anti‐inflammatory properties [5]. NO is synthesised from L‐arginine by the enzyme endothelial nitric oxide synthase (eNOS).

The gene encoding eNOS (*NOS3*) is located on chromosome 7q35‐36 and is a candidate gene for susceptibility to metabolic and vascular diseases. Several single nucleotide polymorphisms (SNPs) have been identified within the *NOS3* gene, with the G894T polymorphism (rs1799983) in exon 7 being one of the most extensively studied. This polymorphism results in a missense mutation, substituting glutamate with aspartate at codon 298 (Glu298Asp) of the protein [5]. This amino acid change is located in a region critical for protein–protein interactions and may alter the enzyme's function. It has been proposed that the Asp298 variant is more susceptible to proteolytic cleavage, leading to reduced eNOS activity and decreased NO bioavailability [4, 6].

Given that NO modulates hepatic and peripheral glucose metabolism as well as insulin secretion [7], a genetically determined reduction in its synthesis could plausibly contribute to the development of insulin resistance and T2D. Therefore, this study was designed to investigate the association between the eNOS G894T polymorphism and the prevalence of T2D in a Tunisian population.

## 2 Materials and Methods

### 2.1 Study Population

A case–control study was conducted between February and November 2014. The study population consisted of 200 unrelated individuals divided into two groups: a case group of 100 patients diagnosed with T2D, and a control group of 100 non‐diabetic volunteers. All participants were recruited from the endocrinology and biochemistry departments of the Military Hospital of Tunis. The study was conducted in accordance with ethical guidelines, and informed consent was obtained from all participants. The case group consisted of individuals with type 2 diabetes, aged ≥ 18 years, hospitalised in the Endocrinology and Diabetology Department of the Military Hospital. Hypertensive and dyslipidemic diabetics were included in this study.

In the control group, apparently healthy non‐diabetic subjects not receiving any treatment were included in the study. Controls were recruited from medical staff and volunteers.

A careful investigation of all the participants was carried out in order to detect all undeclared or unknown pathologies with matching in age and sex between the two groups.

For the final analysis, complete data were available for 69 T2D patients and 71 controls.

### 2.2 Clinical and Biochemical Analyses

For each participant, anthropometric data and clinical history, including family history of metabolic diseases, were collected via a standardised questionnaire. Fasting blood samples (12 h) were collected.

- Lipid profile: Total cholesterol (TC), triglycerides (TG) and high‐density lipoprotein cholesterol (HDL‐C) were measured using enzymatic colorimetric methods on a Unicell DXC800 Beckman Coulter analyser. Low‐density lipoprotein cholesterol (LDL‐C) was calculated using the Friedewald formula. Apolipoproteins A1 (ApoA1) and B (ApoB) were quantified by immunonephelometry on a BNII Siemens analyser.
- Glycemic control: Glycated haemoglobin (HbA1c) was measured for diabetic subjects using High‐Performance Liquid Chromatography (HPLC) on an ADAMS A1C HA8180V analyser.
- Inflammatory marker: High‐sensitivity C‐reactive protein (CRPus) was measured by immunonephelometry.

According to the American Diabetes Association (ADA) and the WHO, the diagnosis of T2D can be established in different ways:

– A1C ≥ 6.5% (≥ 48 mmol/mol).
– Symptoms of diabetes (polyuria, polydipsia, unexplained weight loss, drowsiness or even coma) and blood glucose levels at any time ≥ 2.00 g/L (11.1 mmol/L).
– Fasting blood glucose (FPG) levels ≥ 1.26 g/L (7.00 mmol/L), in the absence of unequivocal hyperglycemia, diagnosis requires two abnormal results from different tests which may be obtained at the same time (e.g., A1C and FPG), or the same test at two different time points.
– Blood glucose levels 2 h after a 75 g glucose load during an oral glucose tolerance test > = 2.00 g/L (11.1 mmol/L).

### 2.3 Genetic Analysis

Genomic DNA was extracted from peripheral blood leukocytes collected in EDTA tubes, using a standard salting‐out method. The purity and concentration of DNA were assessed by measuring absorbance at 260/280 nm.

The G894T polymorphism was genotyped using PCR‐RFLP. A DNA fragment of 248 bp encompassing the polymorphic site was amplified using the following primers:

- Forward: 5′‐AAGGCAGGAGACAGTGGATGGA‐3′
- Reverse: 5′‐CCCAGTCAATCCTTTGGTGCTCA‐3′

PCR was performed in a 50 μL reaction volume. The amplification protocol consisted of an initial denaturation at 94°C for 2 min, followed by 30 cycles of denaturation at 94°C for 20 s, annealing at 55.2°C for 20 s and extension at 72°C for 30 s, with a final extension at 72°C for 5 min.

The 248 bp PCR product was then digested with the *Ban*II restriction enzyme at 37°C for 16 h. The G‐to‐T substitution creates a recognition site for *Ban*II. The resulting fragments were separated by electrophoresis on a 1.7% agarose gel and visualised. The genotypes were determined as follows:

- GG (wild‐type): One uncut band of 248 bp.
- TT (homozygous mutant): Two bands of 163 and 85 bp.
- GT (heterozygous): Three bands of 248, 163 and 85 bp.

### 2.4 Statistical Analysis

Statistical analysis was performed using SPSS version 19.0. Continuous variables were expressed as mean ± standard deviation (SD) and compared using Student's *t*‐test. Categorical variables were compared using the Chi‐squared (*χ* ^2^) test. Hardy–Weinberg equilibrium was assessed in the control group. Odds Ratios (OR) with 95% confidence intervals (CI) were calculated to estimate the risk associated with specific genotypes and alleles. A *p*‐value < 0.05 was considered statistically significant.

## 3 Results

### 3.1 Anthropometric, Clinical and Biochemical Characteristics

The characteristics of the T2D patients and control subjects are summarised in Table 1. The groups were comparable in terms of age and BMI. However, the T2D group had a significantly higher prevalence of hypertension (HTA), dyslipidemia, alcohol consumption and a family history of diabetes, HTA and dyslipidemia.

**TABLE 1:** Clinical and biochemical characteristics of T2D patients and controls.

Parameter (mean ± SD or percentage [%])	Controls (*n* = 71)	T2D patients (*n* = 69)	*p*
Age (years)	49.35 ± 8.96	51.41 ± 6.48	0.122
Sex ratio (M/F)	2.94	1.37	0.037
BMI (kg/m^2^)	28.45 ± 4.28	29.41 ± 5.50	0.257
HTA (%)	0	46.37	< 0.001
Dyslipidemia (%)	0	62.32	< 0.001
Alcohol consumption (%)	4.22	21.73	0.002
Family history diabetes (%)	44.68	79.41	< 0.001
TC (mmol/L)	4.43 ± 0.64	4.63 ± 1.24	0.235
TG (mmol/L)	1.45 ± 0.65	1.99 ± 1.27	**0.002**
HDL‐C (mmol/L)	1.05 ± 0.30	1.02 ± 0.28	0.650
LDL‐C (mmol/L)	2.68 ± 0.63	2.71 ± 0.63	0.836
CRPus (mg/L)	1.63 ± 1.42	2.73 ± 2.47	**0.003**

*Note:* Bold values considered statistically significant at *p* < 0.05.

Biochemically, T2D patients showed significantly higher levels of triglycerides and CRPus compared to controls. No significant differences were observed for TC, HDL‐C, LDL‐C, ApoA or ApoB levels.

### 3.2 Genotype and Allele Frequencies of eNOS G894T

The genotype distributions in both groups are presented in Table 2. The control group was in Hardy–Weinberg equilibrium.

**TABLE 2:** Genotype and allele frequencies of the eNOS G894T polymorphism.

Frequency	Controls (*n* = 71)	T2D patients (*n* = 69)	*p*	OR (95% CI)
Genotypes				
GG (%)	77.46	44.93	*p* < 0.001	1 (Ref.)
GT (%)	19.72	55.07	*p* < 0.001	—
TT (%)	1.41	0	*p* < 0.001	—
Alleles				
G (%)	87.32	72.47	*p* < 0.001	1 (Ref.)
T (%)	11.27	27.53	*p* < 0.001	**4.495 (2.14–9.44)**

*Note:* Bold values considered statistically significant at *p* < 0.05.

A significant difference was observed in the genotype and allele frequencies between the T2D patients and controls. The frequency of the wild‐type GG genotype was significantly lower in the T2D group (44.93%) compared to the control group (77.46%). Conversely, the heterozygous GT genotype was significantly more prevalent among T2D patients (55.07% vs. 19.72%).

The frequency of the mutant T allele was significantly higher in the T2D group (27.53%) than in the control group (11.27%). The presence of the T allele conferred a 4.5‐fold increased risk of developing T2D (OR = 4.495, 95% CI = 2.14–9.44, *p < 0.001*).

## 4 Discussion

This study investigated the association between the functional eNOS G894T polymorphism and the risk of T2D in a Tunisian cohort. Our primary finding is a strong and significant association, with the T allele and the GT genotype being considerably more frequent in T2D patients than in healthy controls. The presence of the T allele was associated with a nearly 4.5‐fold increased risk of the disease, suggesting it is a potent susceptibility factor in this population.

Our results align with several previous studies. Angeline et al. [3] reported a similar high frequency of the GT genotype (55%) in a South Indian diabetic population and found a significant association between the T allele and T2D (OR = 5.289). Similarly, studies in Chinese and Caucasian populations have identified the T allele as a risk factor for hyperglycemia and T2D [7, 8]. A meta‐analysis by Jia et al. [9] also concluded that the G894T polymorphism is significantly associated with an increased risk of T2D.

However, the literature is not entirely consistent. Studies by De Syllos et al. [10] and Santos et al. did not find a significant difference in genotype or allele distributions between T2D patients and controls. These discrepancies may be attributed to several factors, including ethnic and genetic background variations, differences in sample size and potential gene–environment interactions [11]. For instance, Bressler et al. [12] found an association between G894T and T2D only in obese individuals, highlighting the interplay between genetic predisposition and environmental risk factors.

From a biochemical standpoint, the G894T polymorphism's impact on T2D risk is biologically plausible. The Glu298Asp substitution occurs within a domain of the eNOS enzyme that is susceptible to post‐translational modification and cleavage. It has been demonstrated that the Asp298 variant undergoes increased proteolytic cleavage, resulting in lower levels of functional, full‐length eNOS protein [4]. This leads to reduced NO synthesis, impaired endothelial function and an increase in oxidative stress [13]. NO plays a crucial role in insulin signalling by promoting blood flow to skeletal muscle, thereby facilitating glucose uptake [14, 15]. A reduction in NO bioavailability can therefore directly contribute to insulin resistance, a cornerstone of T2D pathophysiology.

In addition to our genetic findings, our study confirmed that T2D is associated with a pro‐inflammatory and dyslipidemic state. The significantly elevated levels of CRPus in our patient group reflect the chronic low‐grade inflammation that characterizes T2D and contributes to insulin resistance [16]. The higher triglyceride levels are also a hallmark of diabetic dyslipidemia, often linked to hepatic insulin resistance and increased VLDL production. These metabolic disturbances are exacerbated by endothelial dysfunction, creating a vicious cycle that accelerates vascular complications.

Limitations of our study include the relatively modest sample size. The lack of verification of ethnicity and consanguinity between participants also represented a limitation of our study. Nevertheless, the strength of the observed association and its biological plausibility provide compelling evidence for the role of the eNOS G894T polymorphism in T2D pathogenesis.

## 5 Conclusion

In conclusion, this study demonstrates a strong association between the eNOS G894T gene polymorphism and Type 2 Diabetes in the studied Tunisian population. The T allele is a significant risk factor, likely contributing to disease susceptibility by reducing eNOS activity, decreasing NO bioavailability and promoting the endothelial dysfunction and insulin resistance that underpin T2D. Further large‐scale and multi‐ethnic studies are warranted to confirm this association and to explore its utility in risk stratification and personalised therapeutic strategies for T2D

## Author Contributions

Conceptualization: Afif Ba, Mariem Belhedi, Manel Ayoub and Sana Aboulkacem. Methodology: Afif Ba and Manel Ayoub. Investigation: Afif Ba. Data curation and analysis: Afif Ba and Manel Ayoub. Writing – original draft: Afif Ba, Mariem Machghoul and Manel Ayoub. Writing – review and editing: Afif Ba and Mariem Belhedi. Supervision: Sana Aboulkacem and Chakib Mazigh. Project administration: Afif Ba and Zied Aouni.

## Conflicts of Interest

The authors declare no conflicts of interest.

## Data Availability

The authors have nothing to report.
